# Response to: Pre-referral rectal artesunate in severe malaria: a flawed trial

**DOI:** 10.1186/1745-6215-12-189

**Published:** 2011-08-08

**Authors:** Melba F Gomes

**Affiliations:** 1 UNICEF/UNDP/World Bank/WHO Special Programme for Research and Training in Tropical Diseases, Geneva, Switzerland

## Abstract

A response to and comment on **Pre-referral rectal artesunate in severe malaria: a flawed trial**, by Karim F Hirji and Zulfiqarali G Premji.

## Letter

The most serious of the criticisms raised by Professors Hirji and Premji in their assessment [1] of our study [2] is that patients might have been enrolled by inadequately trained village health workers and that patient safety and welfare may therefore have been compromised. This is speculative, and inaccurate. In the design of the study and in its implementation, scrupulous care was taken to ensure that all patients were carefully assessed, treated and referred to facilities according to best practice in the region and country, applying of course to both placebo and artesunate recipients. It was a condition of the study that sites should benefit from capacity development in the conduct of clinical trials, strengthening of village health worker involvement in the community, and public participation and education at village level.

The design, execution and analysis reflected the two principal outcomes of death or permanent disability which were clearly defined. The study was planned to allow for differences in practice and malaria endemicity at the various study sites, thus making it possible to reach more general conclusions based on large numbers and variable conditions of practice. Interim analyses were conducted by an independent data monitoring committee, the outcomes of which were not communicated during the course of the study to the investigators. A data analysis plan agreed before unblinding defined the exclusion of patients who did not have malaria or had already received parenteral anti-malarial just before randomisation. In the event, our study produced significant and clinically meaningful results.

There was exceptionally high level of follow up and independent monitoring of the study, including continuing peer review, which confirmed the scrupulous attention to detail and accuracy of the study at all its sites and in all aspects. At study termination the trial was inspected by international regulatory inspectors (hosted by national regulatory authorities) to determine that the rights, safety and welfare of human subjects had been protected, verify procedures (including quality control and training) and the validity of the data; each inspection concluded positively in every aspect.

I cannot comment on Hirji and Premji's analysis of our data using other statistical methods, based on data that by their own submission appeared to be insufficient; nor on what they arbitrarily refer to as 'unlikely outcomes'.

## Competing interests

The authors declare that they have no competing interests.
